# The Link Between Endoplasmic Reticulum Stress and Lysosomal Dysfunction Under Oxidative Stress in Cancer Cells

**DOI:** 10.3390/biom15070930

**Published:** 2025-06-25

**Authors:** Mariapia Vietri, Maria Rosaria Miranda, Giuseppina Amodio, Tania Ciaglia, Alessia Bertamino, Pietro Campiglia, Paolo Remondelli, Vincenzo Vestuto, Ornella Moltedo

**Affiliations:** 1 Department of Pharmacy, University of Salerno, Via G. Paolo II, 84084 Fisciano, SA, Italy; mvietri@unisa.it (M.V.); mmiranda@unisa.it (M.R.M.); tciaglia@unisa.it (T.C.); abertamino@unisa.it (A.B.); pcampiglia@unisa.it (P.C.); moltedo@unisa.it (O.M.); 2 Department of Medicine, Surgery and Dentistry “Scuola Medica Salernitana”/DIPMED, Via S. Allende, 84081 Baronissi, SA, Italy; gamodio@unisa.it (G.A.); premondelli@unisa.it (P.R.)

**Keywords:** lysosomal dysfunction, oxidative stress, ER stress, autophagy, LMP, cancer

## Abstract

Lysosomal dysfunction and endoplasmic reticulum (ER) stress play essential roles in cancer cell survival, growth, and stress adaptation. Among the various stressors in the tumor microenvironment, oxidative stress (OS) is a central driver that exacerbates both lysosomal and ER dysfunction. In healthy cells, the ER manages protein folding and redox balance, while lysosomes regulate autophagy and degradation. Cancer cells, however, are frequently exposed to elevated levels of reactive oxygen species (ROS), which disrupt protein folding in the ER and damage lysosomal membranes and enzymes, promoting dysfunction. Persistent OS activates the unfolded protein response (UPR) and contributes to lysosomal membrane permeabilization (LMP), leading to pro-survival autophagy or cell death depending on the context and on the modulation of pathways like PERK, IRE1, and ATF6. Cancer cells exploit these pathways by enhancing their tolerance to OS and shifting UPR signaling toward survival. Moreover, lysosomal impairment due to ROS accumulation compromises autophagy, resulting in the buildup of damaged organelles and further amplifying oxidative damage. This vicious cycle of ROS-induced ER stress and lysosomal dysfunction contributes to tumor progression, therapy resistance, and metabolic adaptation. Thus, targeting lysosomal and ER stress responses offers potential as cancer therapy, particularly in increasing oxidative stress and promoting apoptosis. This review explores the interconnected roles of lysosomal dysfunction, ER stress, and OS in cancer, focusing on the mechanisms driving their crosstalk and its implications for tumor progression and therapeutic resistance.

## 1. Introduction

Oxidative stress (OS) occurs when reactive oxygen species (ROS) production exceeds the cellular antioxidant capacity, leading to damage to lipids, proteins, and DNA [1].

Within the endoplasmic reticulum (ER), ROS accumulation worsens protein misfolding and unfolding, triggering ER stress. The ER is particularly susceptible to oxidative damage because protein folding is a redox-sensitive process that requires a controlled environment [2]. The accumulation of misfolded proteins triggers the unfolded protein response (UPR), a signaling pathway that attempts to restore normal ER function by halting protein translation, degrading misfolded proteins, and enhancing the production of molecular chaperones [3,4].

ROS generated in the ER can activate stress-related signaling pathways, leading to further production of ROS, particularly in lysosomes, where they can alter the function of lysosomal enzymes and membranes. Lysosomes are crucial for cellular degradation processes, and their acidity is vital for enzyme activity and pathogen destruction. The lysosomal endocytosis pathway facilitates the degradation of extracellular material through a series of maturation steps. Endocytosed material first reaches early endosomes, where a slightly acidic environment promotes the dissociation of ligands from their receptors, with most components being recycled back to the plasma membrane. Cargo destined for degradation progresses to late endosomes, also known as multivesicular bodies (MVBs), characterized by intraluminal vesicles that enable efficient sorting. This maturation process, regulated by the Rab5 to Rab7 transition, involves the acquisition of lysosomal enzymes from the trans-Golgi network. Late endosomes then undergo fusion with lysosomes, where lysosomal hydrolases, membrane proteins, and proton pumps facilitate the breakdown of macromolecules. OS can directly impact lysosomal function by altering redox-sensitive cysteine residues and affect lysosomal acidity, leading to impaired protein degradation and lysosomal dysfunction. Dysregulated lysosomal activity can contribute to ROS production and OS through various mechanisms, including the activation of pro-inflammatory signaling pathways and induction of mitochondrial dysfunction. Moreover, emerging evidence indicates that ER stress can modulate lysosomal enzymes expression and activity, potentially exacerbating lysosomal dysfunction and cellular OS [5,6].

Although OS is a central driver of ER and lysosomal dysfunction, it is part of a broader landscape of cellular stressors that includes hypoxia, nutrient deprivation, metabolic stress, and genotoxic damage. Tumor microenvironments are frequently characterized by low oxygen and glucose availability, altered mitochondrial function, and the accumulation of misfolded proteins, all of which cooperatively impact ER and lysosomal function.

The purpose of this review is to examine the intricate relationship between lysosomal dysfunction, OS, and ER stress in cancer. First, we provide an overview of lysosomal function and its regulation by ROS, followed by a discussion of lysosomal membrane permeabilization (LMP) and its implications in cancer progression. We then explore the impact of OS on lysosomal integrity and its interplay with autophagy in tumor cells. Finally, we highlight the crosstalk between lysosomal dysfunction and ER stress, emphasizing their collective role in cancer survival and therapeutic resistance. A deeper understanding of these interconnected pathways may uncover novel therapeutic strategies aimed at targeting cancer cell vulnerabilities.

## 2. Lysosomal Dysfunction and OS

Reactive oxygen species (ROS), such as superoxide (O_2_^−^), hydrogen peroxide (H_2_O_2_), and hydroxyl radicals (OH), are produced by mitochondria, ER, peroxisomes, and in response to environmental stressors like UV radiation. The electron transport chain and NADPH oxidases (NOXs) are key contributors to ROS production. ER dysfunction and protein misfolding further increase ROS levels.

While ROS are involved in physiological signaling, their excess causes damage to mitochondria, DNA, and lysosomes.

### 2.1. The Lysosomes: Biogenesis and Modulated Processes

Lysosomes are known for their low pH (pH 4.5–5) and high concentration of acidic enzymes, which allow them to degrade cellular waste and recycle components [7]. These enzymes include a broad repertoire of hydrolases, proteases, nucleases, glycosidases, lipases, and sulfatases, that provide degradative capacity across diverse substrates. Lysosomal enzymes function optimally in the acidic pH environment maintained by the cooperation of an ATP-driven proton pump, called the vacuolar H^+^-adenosine triphosphatase (V-ATPase), with other ion channels, ensuring their specificity and efficiency in substrate degradation. These lysosomal pumps comprise two segments: the V_1_ domain, responsible for irreversible ATP hydrolysis, and the V_0_ domain, facilitating the translocation of H^+^ ions across the lysosomal membrane. The continual movement of protons establishes a positive charge within the lysosomal lumen, achieving a balance between the energy gradient (which expels H^+^ ions from the lysosome) and electrostatic repulsion (which impedes the entry of new H^+^ ions). This equilibrium state ensures that the rate of new H^+^ ion entry equals the rate of exit, thereby maintaining a relatively stable proton concentration inside the lysosome. This internal concentration is approximately one hundred times higher than that found in the cytosol [8]. In addition to hydrolases, lysosomes act as dynamic reservoirs of redox-active ions, including Fe^2+^, Zn^2+^, and Ca^2+^. These ions participate in intracellular signaling pathways and help to regulate OS. Their dysregulation under pathological conditions, such as cancer, can exacerbate ROS generation and contribute to lysosomal and cellular dysfunction in both normal physiology and cancer [9,10]. These proteases are involved in protein turnover, antigen presentation, and extracellular matrix remodeling. Cathepsins, which are among the most abundant lysosomal proteases, are categorized into several families based on their catalytic residues, including cysteine (e.g., cathepsin B, L, and S), aspartic (e.g., cathepsin D), and serine cathepsins. In cancer, their aberrant expression is associated with tumor invasion, angiogenesis, and immune evasion. For instance, cathepsin B is often overexpressed in solid tumors and promotes extracellular matrix degradation [11], while cathepsin D is implicated in hormone receptor-positive breast cancer and has been linked to metastatic potential [12]. In the tumor context, altered expression or activity of specific cathepsin isoforms has been linked to invasion, metastasis, and resistance to cell death. Their dysregulation may influence tumor progression through effects on proteolysis, immune modulation, and extracellular matrix remodeling [13,14].

Lysosomal acidity is vital for enzyme activity, membrane transport, and pathogen destruction. ROS can directly modify cysteine residues on the V-ATPase subunits, leading to conformational changes that enhance the pump’s activity. This modification can promote the assembly of V-ATPase from its cytosolic (V_1_) and membrane-bound (V_0_) subunits, facilitating its functional activation. ROS can activate autophagy modulators, primarily AMP-activated protein kinase (AMPK), which then phosphorylates the V_1_ domain of the V-ATPase, leading to increased pump activity. This phosphorylation enhances the assembly of the V-ATPase complex on the lysosomal membrane, boosting its proton-pumping efficiency. ROS may also influence the trafficking of V-ATPase to lysosomes or other acidic compartments within the cell, ensuring that the enzyme is positioned where it is most needed to regulate pH and degrade cellular components. The hyperactivity of the V-ATPase can excessively acidify the lysosomal lumen, potentially disrupting the delicate balance of pH regulation and ion homeostasis within lysosomes. This imbalance can lead to lysosomal swelling, leakage of lysosomal contents into the cytoplasm, and subsequent cellular damage or apoptosis [15].

Additionally, ROS are involved in activating lysosomal enzymes, facilitating their maturation and catalytic activity within the lysosomal compartment. Furthermore, ROS can serve as signaling molecules, modulating various cellular processes, including lysosomal biogenesis, cell death processes, and especially autophagy [16]. During autophagy, damaged organelles, protein aggregates, and intracellular pathogens are sequestered within double-membrane vesicles called autophagosomes, which subsequently fuse with lysosomes for degradation. Lysosomal enzymes hydrolyze the engulfed cargo of the inner membrane of autophagosomes and the elimination of autophagolysosomes, releasing nutrients for cellular metabolism and maintaining cellular homeostasis [17]. These hybrid structures, also known as autolysosomes, represent the final degradative stage of the autophagic process. Their formation is regulated by specific molecular machinery, including Rab7, a small GTPase involved in late endosome and autophagosome maturation; SNARE proteins, which mediate membrane fusion; and the HOPS complex, a tethering factor that facilitates autophagosome–lysosome docking [18]. In parallel, the transcription factor EB (TFEB), a master regulator of lysosomal biogenesis and autophagy, enhances the expression of genes required for autolysosome formation in response to oxidative or nutrient stress [19]. Unlike conventional lysosomes, which primarily degrade material of endocytic origin, autolysosomes are specialized for intracellular recycling and undergo dynamic remodeling of their membrane composition and enzymatic content. Under conditions of excessive ER stress or oxidative imbalance, autolysosome formation may be impaired due to defective acidification or membrane fusion, leading to the accumulation of undegraded cargo and disrupted proteostasis [20]. In cancer cells, efficient autolysosome activity supports adaptation to hostile microenvironments by sustaining intracellular clearance and promoting survival during metabolic and therapeutic stress [21]. The process of autolysosome formation is a key feature of macroautophagy, the most studied form of autophagy. However, lysosomes also play central roles in other forms of autophagy, which differ in their mechanisms of cargo recognition and delivery. There are three types of autophagy: macroautophagy, microautophagy, and chaperone-mediated autophagy (CMA). Macroautophagy involves the formation of double-membraned autophagosomes that fuse with lysosomes to form autolysosomes, where cathepsins degrade the autophagic cargo. Microautophagy directly engulfs cytoplasmic constituents or organelles into lysosomes, either selectively or non-selectively. CMA targets specific proteins for degradation via Hsc70, which recognizes the KFERQ motif in substrates and delivers them to the lysosomal lumen via LAMP2A [22]. As mentioned above, moderate levels of ROS can induce autophagy by activating several signaling pathways, including those mediated by AMPK and JNK (c-Jun N-terminal kinase). AMPK is activated in response to changes in cellular energy status, leading to the inhibition of mTOR (mechanistic target of rapamycin), a key negative regulator of autophagy. This inhibition of mTOR removes its suppressive effect on the autophagy-initiating complex ULK1, thus promoting autophagy. Simultaneously, ROS can activate JNK, which phosphorylates and modulates the activity of various autophagy-related proteins, such as Beclin-1. This phosphorylation enhances the formation of the Beclin-1 complex, crucial for the initiation of autophagy. Together, these pathways enable cells to degrade damaged organelles and proteins, maintaining cellular homeostasis and adapting to OS conditions. However, excessive ROS production can impair autophagic flux, leading to autophagy dysfunction and cellular damage [23].

Lysosomal biogenesis, orchestrated by transcription factors like TFEB and MITF (microphthalmia-associated transcription factor), hinges on intricate regulatory mechanisms involving nutrient availability, ROS, and calcium levels [24]. Under stress, TFEB and MITF translocate to the nucleus to activate lysosomal genes via CLEAR elements (coordinated lysosomal expression and regulation elements). In nutrient-rich environments, TFEB and MITF remains inactive due to mTORC1 (mechanistic target of rapamycin complex 1)-mediated phosphorylation at serine residues. Conversely, nutrient starvation or OS inhibits mTORC1, leading to TFEB and MITF dephosphorylation and subsequent nuclear translocation and activation of genes involved in lysosomal function and biogenesis. This ROS-dependent translocation highlights the critical role of TFEB and MITF oxidation in modulating autophagy and lysosomal biogenesis under OS.

#### 2.1.1. TRPML1

Lysosomal membranes contain redox sensors that respond to ROS. One such sensor is the transient receptor potential mucolipin channel 1 (TRPML1), a lysosomal Ca^2+^ channel, which plays a role in autophagy, OS sensing, and lysosomal exocytosis [25].

When ROS levels rise, they can oxidize specific cysteine residues on TRPML1, leading to conformational changes that open the channel and allow Ca^2+^ to exit the lysosome. The released Ca^2+^ activates downstream signaling pathways, such as calcineurin, which dephosphorylates TFEB, promoting its nuclear translocation and inducing genes involved in autophagy and lysosomal biogenesis, like ATG genes (autophagy-related genes) or LAMP1 and LAMP2 (lysosome-associated membrane proteins 1 and 2), critical for maintaining lysosomal integrity and function. This process helps to remove damaged mitochondria and regulate cellular redox homeostasis [26]. When TRPML1 is disabled genetically or suppressed with drugs, the cell’s ability to eliminate damaged mitochondria and reduce excessive ROS is impaired. Moreover, TRPML1′s responsiveness to ROS is crucial for lysosomes to adapt to mitochondrial injury. Therefore, TRPML1 acts as a sensor for ROS positioned on the lysosomal membrane, coordinating a negative-feedback mechanism reliant on autophagy to alleviate OS within the cell [27].

TRPML1 channels also facilitate the transport of Fe^2+^ out of the lysosome into the cytoplasm by releasing Fe^2+^ freed from degraded ferritin, which is transported to lysosomes for degradation, ensuring that lysosomal iron levels are properly regulated [28]. ROS can impact these ion channels, leading to disruptions in lysosomal function. Excessive iron accumulation, for example, can catalyze the formation of toxic granules like lipofuscin, which inhibits lysosomal function and contributes to OS through the Fenton reaction with hydrogen peroxide due to high metal levels contained in lipofuscins [29,30]. Elevated ROS levels can peroxidize lipids in cell membranes, triggering the lipid peroxidation chain reactions that are a hallmark of ferroptosis, a form of regulated cell death characterized by iron-dependent lipid peroxidation amplifying OS [31].

#### 2.1.2. TRPML2

TRMPL2 is a Ca^2+^-permeable nonselective cation channel in the TRP superfamilies and is highly expressed in the brain and peripheral blood cells. TRPM2 is also present on lysosomal membranes in pancreatic β cells, dendritic cells, and cancer cells, where it regulates processes like cell death, maturation, chemotaxis, and migration by mediating Ca^2+^ and Zn^2+^ release [32].

ROS can induce conformational changes in TRPML2, leading to its opening and subsequent ion release. This activation helps in the regulation of lysosomal function and the cellular response to stress, promoting processes like autophagy to clear damaged components and mitigate oxidative damage [33].

#### 2.1.3. TPCs and BK

TPCs (two-pore channels), particularly TPC2 and TPC1, are another class of lysosomal channels involved in releasing Ca^2+^ and Na^+^ ions [34]. ROS can directly oxidize TPCs or their regulatory proteins, enhancing calcium release and contributing to cellular stress responses. ER stress triggers the UPR, which can indirectly affect TPC function by altering lysosomal homeostasis and calcium fluxes, further amplifying autophagy and apoptosis pathways in response to prolonged stress. Sun et al., in their study, observed that overexpressing TPC in 4T1 mouse breast cancer cells and HeLa human cervical cancer cells disrupted autophagosome–lysosome fusion. This led to autophagosome buildup, an increase in lysosomal pH, and more TFEB present in the nucleus. Notably, cells with elevated TPC2 levels showed reduced secretion of extracellular vesicles (EVs), whereas EVs release was enhanced in TPC2-knockdown cells. Additionally, cell migration decreased in TPC2 knockdown cells, but remained unaffected in TPC2-overexpressing cells. These findings highlight TPC2′s influence in modulating autophagy and EVs trafficking in cancer cells, suggesting its potential role in cancer progression [35].

Large conductance Ca^2+^-activated K^+^ channels (BK channels), initially identified on plasma membranes, are influenced by oxidizing and reducing agents. Evidence suggests that the oxidation of cysteines and methionines on BK channels affects their properties. Recent studies have revealed that BK channels also exist on lysosomes, supporting TRPML1 functions and lysosomal Ca^2+^ maintenance [36,37].

#### 2.1.4. TMEM175 and CLC-3

TMEM175, another lysosomal potassium channel, plays a critical role in maintaining lysosomal membrane potential and pH stability by facilitating K^+^ conductance [38]. ROS can alter the redox environment within lysosomes, potentially affecting TMEM175′s conductance properties, although the exact mechanisms are less understood compared with those of TRPML channels. By maintaining the ionic balance within lysosomes, TMEM175 ensures proper lysosomal function and acidification, which are crucial for the degradation of cellular waste and the overall health of the cell [39].

**Figure 1:**
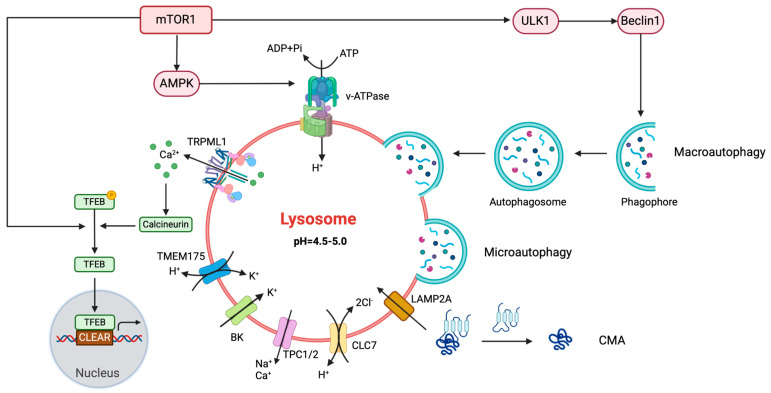
Schematic representation of a functional lysosome, highlighting its enzymatic activities, ion channels, and role in autophagy. Lysosomes maintain an acidic environment (pH 4.5–5) via V-ATPase (vacuolar H^+^-adenosine triphosphatase), ensuring the activation of over 60 hydrolytic enzymes that degrade cellular waste, damaged organelles, and misfolded proteins. Several ion channels regulate lysosomal function: transient receptor potential mucolipin 1 (TRPML1), which releases Ca^2+^ and activates calcineurin to drive the nuclear translocation of transcription factor EB (TFEB); TRPML2, which traffics Ca^2+^/Zn^2+^ and modulates immune responses; two-pore channels 1/2 (TPC1/2), regulating Na^+^ and Ca^2+^ flux; large-conductance Ca^2+^-activated K^+^ (BK) channels, stabilizing membrane potential; transmembrane protein 175 (TMEM175), mediating K^+^ conductance; and chloride channels 3/7 (CLC-3/7), supporting Cl^−^/H^+^ exchange for acidification. Signaling pathways include AMP-activated protein kinase (AMPK), which phosphorylates V-ATPase to boost lysosomal activity, and the mechanistic target of rapamycin complex 1 (mTORC1), which suppresses autophagy via UNC-51-like autophagy-activating kinase 1 (ULK1). Lysosomes coordinate macroautophagy, microautophagy, and chaperone-mediated autophagy (CMA); the latter relies on lysosome-associated membrane protein 2A (LAMP2A) for selective cargo import. Through ion regulation, AMPK/mTORC1/ULK1 signaling, and TFEB activation, lysosomes safeguard cellular homeostasis under stress.

Chloride channels, such as CLC-3, contribute to chloride/proton exchange and help to maintain lysosomal acidity. ROS can modulate their function by altering lysosomal pH and redox balance. Zoledronic acid (ZA), a drug used against bone metastases, induces apoptosis partly through ROS production and chloride channel activation, as shown in studies on nasopharyngeal carcinoma cells [40]. Although CLC-3 is mainly associated with endosomes and not lysosomes, it remains of interest to explore whether lysosomal channels like CLC-7 share similar redox sensitivity (Figure 1).

#### 2.1.5. Lysosomal Membrane Permeabilization (LMP)

A significant consequence of ROS-induced lysosomal dysfunction is LMP, a process in which the integrity of the lysosomal membrane is compromised, leading to the release of lysosomal contents into the cytoplasm [41].

**Figure 2:**
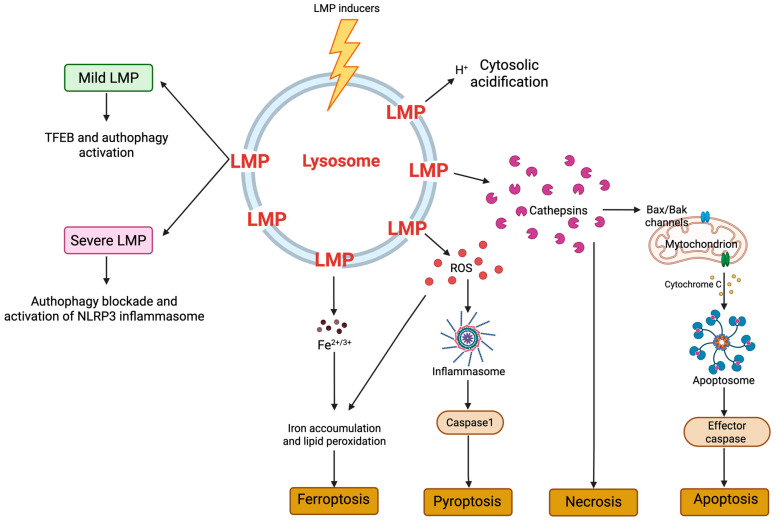
Lysosomal membrane permeabilization (LMP) and its cellular consequences. Oxidative stress and other inducers, such as reactive oxygen species (ROS), lipid peroxidation, calcium overload, and tumor-necrosis-factor-alpha (TNF-α), compromise lysosomal integrity, leading to lysosomal membrane rupture and the release of Fe^2+^ and cathepsins into the cytosol. Free Fe^2+^ catalyzes Fenton reactions, generating excessive ROS and promoting ferroptosis. Meanwhile, cathepsins activate caspase-1 (initiating pyroptosis) and facilitate mitochondrial outer membrane permeabilization to promote B-cell-lymphoma-2-associated X-protein (Bax)/Bax-antagonist killer 1 (Bak) channel opening, leading to cytochrome c release and apoptosome formation, which activates caspase-9 and caspase-3, driving apoptosis. In parallel, mild LMP can activate transcription factor EB (TFEB), promoting lysosomal biogenesis and autophagy. However, severe LMP disrupts autophagosome–lysosome fusion, resulting in autophagy blockade and increased cytotoxicity. LMP also activates the NOD-like-receptor-family pyrin-domain-containing protein 3 (NLRP3) inflammasome, enhancing interleukin-1β (IL-1β) secretion, macrophage activation, and pyroptotic cell death. In some cases, cytosolic acidification and uncontrolled protease activity following LMP can lead to necrosis. Ultimately, the balance between lysosomal repair mechanisms and the activation of death pathways determines cell fate, making LMP a critical regulator in stress responses and disease progression.

The consequences of LMP depend on various factors, including the type and intensity of the stimulus and the extent of lysosomal damage. OS is a primary inducer of LMP. ROS oxidize lysosomal membrane lipids and proteins, increasing membrane permeability. Lipid peroxidation products destabilize the lysosomal membrane, often initiated by enzymes, like lipoxygenases, and ROS [42,43]. The leakage of lysosomal enzymes into the cytoplasm leads to cellular component degradation and activation of cell death pathways, especially under high-OS conditions. This includes apoptosis, where cathepsins activate caspases (e.g., caspase-8 and caspase-3), initiating the apoptotic cascade, and necrosis, where extensive lysosomal damage leads to uncontrolled proteolysis and cell disintegration. LMP also contributes to ferroptosis by releasing iron, which participates in the Fenton reaction, increasing ROS and lipid peroxidation (Figure 2).

Additionally, mild LMP can act as a stress signal that initially triggers autophagy, aiming to recycle damaged lysosomes and other compromised cell components to restore cellular homeostasis. When the lysosomal contents leak, it often stimulates transcription factors, like TFEB, that upregulate lysosomal biogenesis and autophagy-related genes to help the cell to adapt. However, if LMP progresses beyond a certain threshold, it disrupts lysosomal function and impairs the fusion of autophagosomes with lysosomes. This inhibition leads to an accumulation of autophagosomes and prevents the degradation phase of autophagy, effectively blocking the process, indicated by increased LC3-II levels and acidic vacuoles, affecting critical signaling pathways, and leading to cellular conditions that favor cell death over autophagy, due to the buildup of toxic cellular waste and ROS [44].

LMP can also activate the NRLP3 inflammasome, leading to the release of pro-inflammatory cytokines like IL-1β, which subsequently activates macrophages and microglia, and ultimately results in pyroptosis and cell death [45]. Multiple cathepsins are involved in NLRP3 inflammasome activation and in the literature, the involvement of cathepsin B is largely reported [46]. Although it remains uncertain whether LMP and cytoplasmic cathepsin release are directly responsible, LMP has been observed in an in vivo model of coronary arthritis, where silencing cathepsin B has been shown to reduce inflammasome activation [47].

Furthermore, calcium overload triggers LMP through the activation of phospholipases A2 (PLA2) and proteases, like calpains, which cleave proteins that normally maintain lysosomal stability [48]. Several signaling pathways are involved, including the p53 pathway, which induces LMP through pro-apoptotic proteins and mitochondrial effects, and the Bcl-2 family proteins, where pro-apoptotic members (e.g., Bax and Bak) induce LMP, whereas anti-apoptotic members (e.g., Bcl-2 and Bcl-xL) inhibit it [49]. The TNF-α pathway induces LMP through acid sphingomyelinase (ASM) activation, generating ceramide, which disrupts lysosomal membranes through its ability to alter the biophysical properties of the lipid bilayer because ceramide can induce the formation of rigid microdomains, reducing membrane fluidity and increasing its permeability. The involvement of proteins like Hsp70 and membrane components such as sphingomyelin in maintaining lysosomal integrity highlights the complexity of the mechanisms regulating LMP and its significant impact on cellular health and disease [50,51].

## 3. Lysosomal Dysfunction and OS in Cancer Cells

Cancer cells are known to upregulate their metabolism to support growth and proliferation, partly by manipulating lysosomal function. This is achieved by altering the quantity, localization, and activity of lysosomes to enhance the degradation and recycling of macromolecules. The overexpression of lysosomal enzymes (e.g., catalase and glycosidases) and kinesins is observed in several cancers (e.g., pancreatic, renal, melanoma, and breast), making lysosomes promising therapeutic targets [52,53].

Lysosomal dysfunction contributes to OS in cancer cells by disrupting the degradation of damaged organelles, such as mitochondria. Impaired autophagy and mitophagy lead to the accumulation of dysfunctional mitochondria, further increasing ROS production. While enhancing autophagy may reduce ROS and sensitize cells to therapy, inhibiting autophagy can block cancer cell survival under stress. Targeting the mTOR pathway, a central regulator of lysosomal function, may disrupt these adaptive mechanisms [54]. Therefore, understanding the autophagic status of a tumor is crucial for determining whether to inhibit or potentiate autophagy for optimal therapeutic outcomes.

Lysosomes also contribute to chemoresistance by sequestering drugs and the redistribution of lysosomes to the periphery of cancer cells and their exocytosis of enzymes, like cathepsins, heparinase, and Neu1, further promote cancer invasion, metastasis, and angiogenesis by degrading the extracellular matrix and basement membrane [55].

Additionally, cancer cells possess larger and more vulnerable lysosomes compared with normal cells, and cancer cells depend heavily on lysosomes for their proliferation, metabolism, and adaptation to stress, more so than healthy cells [56]. Tumor cells can also boost lysosome production, impacting their quantity. Unlike genetic mutations, cancer cells cannot modify their lysosomes, making these organelles prime targets for innovative anticancer therapies. Consequently, inducing lysosomal cell death offers an alternative approach to eradicate tumor cells that have developed resistance to conventional chemotherapy.

Targeting lysosomes and regulating ROS levels in cancer cells presents a promising avenue for developing innovative cancer therapies. The intricate crosstalk between lysosomal dysfunction and OS offers a crucial yet challenging target for intervention. Understanding these mechanisms, particularly the roles of autophagy and LMP in cancer, could pave the way for more precise and effective therapeutic strategies, ultimately improving patient outcomes.

### 3.1. Autophagy and Cancer

Basal autophagy operates continuously under standard conditions and can be further triggered by various physiological stimuli, including hypoxia, nutrient deprivation, ER stress, energy depletion, hormonal stimulation, and pharmacological interventions. In cancer, autophagy is a double-edged sword: initially tumor-suppressive by removing damaged proteins and organelles, limiting ROS and mutations; but later tumor-promoting, providing nutrients and resistance to therapies [57]. mTOR is central in this switch, regulating both catabolism and anabolism, and mTOR inhibitors are commonly used in cancer treatment, such as rapamycin and its analogs, collectively known as rapalogs, and ATP-competitive mTOR kinase inhibitors, which are designed to target ATP binding sites in the catalytic domains of mTOR. Rapalogs initially bind to the intracellular receptor FK506 binding protein 12 (FKBP12). This complex then interacts with a domain separate from the catalytic site of mTOR, thereby inhibiting mTOR’s function. Rapalogs selectively target mTORC1 and have shown effectiveness as anticancer agents in various preclinical models [58].

Additionally, targeting PI3K (phosphoinositide 3-kinase) and Akt (protein kinase B), which are involved in mTOR activation, is another approach. The PI3K/Akt/mTOR pathway is frequently dysregulated in cancer, leading to uncontrolled cell proliferation, enhanced survival, and resistance to therapies. Activated by growth factors via receptor tyrosine kinases (RTKs), PI3K converts PIP2 to PIP3, recruiting and activating Akt. Akt inhibits TSC2, activating mTORC1, which drives protein synthesis, cell growth, and metabolic adaptation. Persistent Akt activity suppresses apoptosis and contributes to chemotherapy resistance. PTEN acts as a negative regulator by converting PIP3 back to PIP2, limiting pathway activation. Due to its central role in tumor progression, this pathway is a major target for anticancer therapies [59].

As one of the key effector nodes in the PI3K pathway, AKT could be a promising target in PI3K pathway-activated tumors. Pan-AKT inhibitors currently being developed include allosteric inhibitors like MK-2206 and ATP-competitive inhibitors such as AZD5363 and ipatasertib (GDC-0068). Early phase I trials have shown initial efficacy for MK-2206, AZD5363, and ipatasertib, leading to their evaluation across various solid tumors [60,61,62]. Pictilisib (GDC-0941) is under investigation for its potential in treating HER2-positive metastatic breast cancer and advanced non-small-cell lung cancer (NSCLC). It has shown balanced inhibitory effects on PI3K in vitro and phase I clinical trials have revealed preliminary indications of its effectiveness in advanced solid tumors [63].

Wu et al., in their study, highlight the potential of targeting the PI3K/Akt/mTOR pathway in ovarian cancer, particularly in high-stage cases with dysregulated signaling, through preclinical testing in genetically engineered mouse models (GEMs). Using an ovarian epithelial adenocarcinoma (OEA) mouse model with inactivated APC and PTEN genes, researchers tested Akt and mTOR inhibitors, showing efficacy both in vitro and in vivo. Their study also underscores the benefits of GEM models over traditional cell cultures for evaluating drug combinations that could maximize tumor inhibition, while identifying dose-limiting toxicities, laying the groundwork for optimized treatment strategies in clinical trials [64].

Targeting autophagy might enhance sensitivity to treatments, but it also requires careful consideration due to its complex role in cancer. While some studies have shown that targeted inhibition of autophagy can enhance the sensitivity of cancer cells to conventional therapies, including chemotherapy, radiotherapy, and targeted therapy, other studies suggest that autophagy may also contribute to cancer cell survival and drug resistance. Therefore, the safety and efficacy of autophagy-targeted therapies require further experimental validation.

### 3.2. LMP and Cancer

LMP in cancer cells plays a dual role in tumor progression and therapy. On one hand, lysosomal enzymes released through LMP degrade extracellular matrix components and basement membranes, facilitating metastasis and promoting angiogenesis. LMP-induced autophagy also helps tumor cells to survive in hostile environments by recycling intracellular components. On the other hand, LMP can be exploited therapeutically to trigger alternative, caspase-independent cell death pathways in tumors resistant to apoptosis [65].

Conversely, inhibiting LMP can prevent the release of destructive enzymes that aid metastasis and protect normal tissues. Controlled inhibition supports crucial anti-tumor processes like antigen presentation, but a prolonged LMP blockade may inadvertently promote therapy resistance. Many cancer cells overexpress heat shock proteins like HSP70, which stabilize lysosomal membranes and prevent LMP-induced cell death, further enhancing resistance. Targeting HSP70, for instance through inhibitors that disrupt lysosomal membrane stability, has shown promise, particularly in tumors with downregulated ASM expression (e.g., gastrointestinal, hepatocellular, and renal cancers) [66].

Apoptozole, an ATP-competitive HSP70 inhibitor, has shown toxicity against a variety of cancer cells, including those from oral squamous cell carcinoma, breast, and liver cancer. In xenograft models of lung adenocarcinoma, cervical cancer, and colorectal carcinoma, apoptozole effectively suppressed tumor growth. Mechanistic studies have revealed that apoptozole induces caspase-dependent apoptosis by blocking the interaction of HSP70 with APAF-1. Additionally, Park et al. found that apoptozole promotes lysosome-mediated apoptosis and disrupts autophagy in cancer cells [67].

Some anticancer drug precursors are designed to release ROS within lysosomes, exploiting the high levels of free radicals to kill cancer cells [68]. However, the complexity of ROS in cancer necessitates more precise approaches to avoid adverse effects.

Thus, like autophagy, LMP serves as a double-edged sword in cancer therapy. Modulating LMP could provide a targeted strategy: activating it to induce cancer cell death or inhibiting it to prevent unwanted tumor progression, depending on the cancer type and therapeutic goals.

Several substances have been identified that can induce LMP and are being explored as cancer therapies. The anti-CD38 monoclonal antibody SAR650984, for example, has shown promise in combination with pomalidomide, an immunomodulatory drug, for treating multiple myeloma. This compound induces LMP by increasing lysosomal size and triggering membrane permeabilization, effectively killing cancer cells, including those resistant to pomalidomide. SAR650984′s effectiveness is linked to actin cytoskeleton polymerization, leading to high cell membrane CD38 and inter-cellular hypercrosslinking (HA), which induces lysosome-mediated and caspase-dependent cell death. Additionally, this drug triggers ROS production, further contributing to cell death [69].

Sigma-2 receptor ligands, such as siramesine, have shown promising potential in targeting cancer cells, particularly in inducing LMP and triggering cell death. Siramesine, initially explored for anxiety and depression, acts as a lysosomotropic detergent that raises lysosomal pH, leading to LMP and caspase-independent apoptosis. This mechanism has been further validated in preclinical studies on pancreatic cancer models, where sigma-2 ligands like SW43 and PB282 demonstrated efficacy in reducing tumor burden. The compounds accumulate in lysosomes, where they destabilize lysosomal membranes, releasing cathepsins and inducing OS. Interestingly, SW43 relies heavily on OS, with antioxidants like N-acetylcysteine providing cellular protection, whereas PB282-induced apoptosis is more caspase-dependent. These findings underscore sigma-2 ligands as promising therapeutic agents for cancers resistant to standard therapies, with each ligand’s efficacy potentially dependent on its specific apoptotic mechanism [70,71].

In summary, while LMP typically leads to cell death by releasing lysosomal enzymes, it can also promote cancer cell survival through mechanisms such as enhanced tumor invasion, chemoresistance, and stress adaptation. These dual roles of LMP highlight the complexity of its impact on cancer cells and underscore the need for a nuanced understanding of LMP in cancer therapy.

## 4. Lysosomal Dysfunction and ER Stress

The ER is essential for protein quality control, lipid and cholesterol synthesis, calcium storage, and signaling. This is critical for cellular survival, as an imbalance in this process is known to lead to various diseases, including metabolic, neurodegenerative, oncological, and cardiovascular disorders. Structural alterations, calcium imbalance, or OS can impair ER function, leading to the accumulation of misfolded proteins, which disrupts the balance between new protein synthesis and the ER’s capacity to process them, resulting in a state known as ER stress. To restore homeostasis, cells activate an integrated cascade of intracellular signals known as UPR [72,73].

ER stress can activate all branches of the UPR signaling, including both protective and pro-apoptotic pathways. On one hand, the UPR restores cellular homeostasis by upregulating the expression of pro-adaptive genes necessary to transmit signals that enable cell survival during stress conditions. However, if the increase in protein levels exceeds the capacity to restore homeostasis, ER stress persists, and stressed cells are destined for death. Downstream effects of ER stress can impact processes such as ER-associated protein degradation (ERAD), protein synthesis, OS, mitochondrial dysfunction, lysosomal alterations, and autophagy. The latter has been less characterized in the context of ER stress and its subsequent UPR response [74].

The UPR is regulated by three ER sensors: inositol-requiring enzyme 1 (IRE1), ER-resident kinase PERK (PKR-like endoplasmic reticulum kinase), and activating transcription factor 6 (ATF6). These sensors remain inactive due to their binding with BiP. It is believed that unfolded proteins compete with BiP, causing the activation of the three sensors upon BiP dissociation [75].

Each of the three transducers follows a distinct pathway:

(1) PERK initiates the phosphorylation of eukaryotic initiation factor 2 alpha (eIF2α), a subunit of the eukaryotic initiation factor 2 (eIF2) complex, which is essential for the initiation of translation forming a ternary complex with GTP and the initiator tRNA, necessary for the assembly of the ribosome on mRNA and the start of protein synthesis [76]. However, under these conditions, the translation of activating transcription factor 4 (ATF4) mRNA is promoted, further reducing protein accumulation in the ER. ATF4, in turn, under prolonged stress, interacts with C/EBP homologous protein (CHOP), which regulates the expression of target genes such as growth arrest and DNA damage-inducible protein 34 (GADD34) and endoplasmic reticulum oxidoreductin-1 alpha (ERO-1α), and pro-apoptotic factors, including DR5, TRB3, CAV1, and BCL2 family proteins [77]. GADD34 encodes a regulatory subunit of a phosphatase complex that dephosphorylates eIF2α, contributing to protein overload. ERO1α is an enzyme localized in the endoplasmic reticulum that plays a crucial role in oxidative protein folding, which promotes ER hyperoxidation. PERK also regulates several transcription factors, including NRF2, which upregulates the antioxidant response. Under basal conditions, NRF2 is kept inactive by Kelch-like ECH-associated protein 1 (KEAP1), which induces its degradation through the cullin3/ring box 1-dependent ubiquitin ligase complex. In the case of OS, ROS react with specific KEAP1 cysteines, inducing conformational changes that prevent the binding of NRF2, thus freeing NRF2 to migrate into the nucleus. PERK may also induce the phosphorylation and dissociation of NRF2 from KEAP1, enhancing its antioxidant activity. In the nucleus, NRF2 regulates the inducible expression of genes containing antioxidant response elements (AREs), thereby activating the expression of detoxifying enzymes, including NAD(P)H oxidoreductase 1 (NQO1), heme oxygenase 1 (HO-1), glutathione S-transferase (GST), and the rate-limiting enzyme in glutathione biosynthesis, γ-glutamylcysteine synthetase (GCLC). Additionally, both an endogenous anti-inflammatory molecule, 15-deoxy-Δ 12,14 -prostaglandin J2 (15d-PGJ2), and nitric oxide (NO), which plays a role in vasodilation and inflammation, can activate NRF2 [78]. Interestingly, both NO signaling and 15d-PGJ2 have recently been implicated in ER stress signaling, suggesting that NRF2 might also participate in ER stress signaling [79].
(2) IRE1 kinase undergoes oligomerization and autophosphorylation, becoming endo-RNase. In this way, it can excise a 26-nucleotide intron from X-box binding protein 1 (XBP1) mRNA, forming the spliced version known as XBP1s. This spliced form of XBP1 modulates the expression of various UPR target genes involved in protein folding, glycosylation, and ERAD. The endo-RNase activity of IRE1 can affect both mRNA and microRNA, causing regulated IRE1-dependent decay (RIDD). RIDD has emerged as a new regulatory component of the UPR that determines cellular fate during ER stress. Furthermore, during prolonged ER stress, IRE1 can promote the accumulation of CHOP to induce cell death and serves as a scaffold for recruiting TNF receptor-associated factor 2 (TRAF2) and c-Jun N-terminal inhibitory kinase (JIK) to the ER membrane, which subsequently activates the JNK-mediated pathway. The activation of JNK influences cytochrome c release through the phosphorylation of BCL2 family proteins, including BCL2 and BIM, which subsequently promote apoptosis [80,81].
(3) ATF6, during UPR, is transported to the Golgi apparatus, where it is cleaved by the proteases S1P and S2P. This cleavage releases a 50 kDa cytosolic fragment containing the b-ZIP region (ATF6f) that migrates to the nucleus and directly regulates genes responsible for ERAD components, such as bZip family members and XBP1. When ATF6 enters the nucleus, ATF6 becomes part of a multiprotein complex that will bind to mammalian endoplasmic reticulum stress response elements (ERSEs), which are present in the promoters of the UPR target genes. The stimulatory activity of ATF6 depends precisely on the integrity of the ERSE structure, particularly the transcription factor NF-Y, which is required for the docking of ATF6 into the promoters of the UPR target genes [82,83].

It has been shown that excessive ER stress, induced by Brefeldin A (BFA) or tunicamycin, impairs lysosomal function by inhibiting autophagic flux through an increase in autophagosomes and a decrease in autolysosomes. Additionally, the expression levels of LAMP1 and beta-galactosidase (b-gal), a lysosomal hydrolase enzyme, were significantly reduced in the sera of preeclamptic patients compared with normal pregnant women. This suggests that LAMP1 or b-gal in the serum may be used as an indicator of lysosomal damage caused by ER stress. Furthermore, excessive ER stress reduces lysosomal biogenesis, accumulates SQSTM1, and inhibits the fusion of lysosomes and autophagosomes, thus blocking autophagy in trophoblasts. ER stress and compromised autophagy work together to disrupt trophoblastic homeostasis, contributing to preeclampsia and fetal growth restriction (FGR) [84].

Experiments on PERK-knockout mouse embryonic fibroblasts (MEFs) show reduced survival under hypoxia. Additionally, PERK appears to play a crucial role in autophagy, which is increased by stressors such as hypoxia and nutrient deprivation. The activation of key autophagy genes, ATG5 and MAP1, is linked to PERK-dependent signaling through ATF4 and CHOP. The inhibition of the PERK–eIF2α–ATF4 axis reduces autophagy, making cells more sensitive to hypoxia [85].

Adolph et al. demonstrated that autophagy and UPR act complementarily to maintain intestinal homeostasis [86]. In Xbp1-deficient intestinal epithelial cell (IEC) models, a significant increase in the phosphorylation of PERK and its substrate eIF2α was observed, confirming that PERK is responsible for the formation of phosphorylated eIF2α. The presence of ATF4 and CHOP, effectors of the PERK-eIF2α signaling pathway, was increased in these cells, with greater association of ATF4 with the promoters of LC3b and Atg7, key proteins in the autophagy process. Silencing PERK prevented the increase in Atg7. The same result was confirmed in Xbp1-deficient mice. When UPR-induced autophagy was absent, severe intestinal inflammation occurred, like that observed in human Crohn’s disease (CD). Therefore, PERK-p-eIF2α is crucial for UPR-induced autophagy in intestinal cells following Xbp1 deficiency, and the absence of ATG7 leads to significant deterioration of intestinal inflammation, which could clearly, in a chronic manner, lead to the onset of tumor forms.

**Figure 3:**
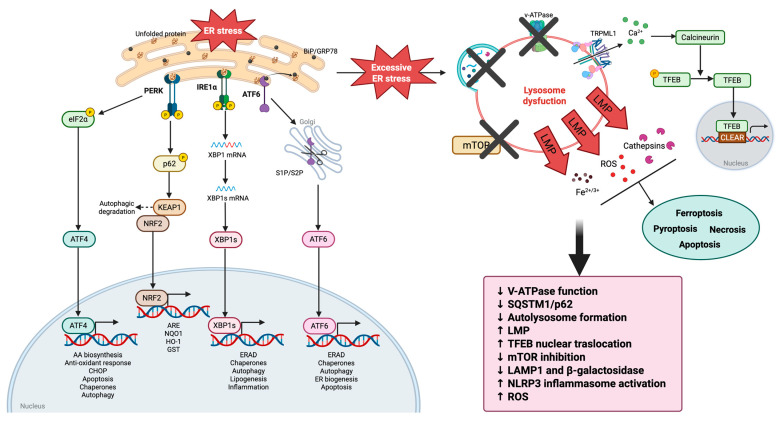
Endoplasmic reticulum (ER) stress and lysosomal dysfunction. ER stress activates the unfolded protein response (UPR) via protein-kinase-RNA-like ER kinase (PERK), inositol-requiring enzyme 1 (IRE1), and activating transcription factor 6 (ATF6). PERK phosphorylates eukaryotic-initiation-factor 2-alpha (eIF2α), up-regulating activating transcription factor 4 (ATF4), which drives the transcription of autophagy genes (autophagy-related proteins, ATG; microtubule-associated-proteins 1A/1B light-chain 3, LC3) and promotes transcriptional factor EB (TFEB) nuclear import through the phosphorylation of sequestosome 1 (SQSTM1/p62). IRE1 splices X-box-binding-protein 1 (XBP1) mRNA, inducing Beclin-1 and enhancing autophagy/lysosomal biogenesis. Cleaved ATF6 up-regulates ER chaperones, further supporting autophagy. Excess ER stress impairs the vacuolar H^+^-adenosine-triphosphatase (V-ATPase), raising lysosomal pH and diminishing lysosome-associated membrane protein 1 (LAMP1) and β-galactosidase (β-Gal), hallmarks of defective autolysosome formation. Accumulated reactive oxygen species (ROS) amplify oxidative damage, while NOD-like-receptor-family pyrin-domain-containing protein 3 (NLRP3) inflammasome activation fosters inflammation and pyroptosis. Prolonged stress also provokes lysosome membrane permeabilization (LMP), releasing cathepsins, ROS, and Fe^2+^ to promote apoptotic, necrotic, and ferroptotic pathways. These interconnected mechanisms underscore how dysregulated UPR signaling converges on lysosomal dysfunction to influence cancer cell survival and death. The × symbol indicates inhibition, impairment, or blockade of the corresponding cellular process under conditions of prolonged stress.

In addition, LMP can be triggered or amplified by a wide range of stimuli, including ER stress, and is involved in certain types of cancer. For example, Chiarante et al. reported that the lipophilic phthalocyanine Pc9, a potent photosensitizer, induces a rapid apoptotic response in murine colon carcinoma cells (CT26) through cooperation between the lysosomal pathway and the ER [87]. Indeed, massive ROS production alters LMP, leading to an increase in cytosolic cathepsin D, a lysosomal enzyme involved in regulating an apoptotic response, thereby activating caspase 8 and contributing to Bid cleavage. Simultaneously, Pc9 photoactivation in the ER activates the UPR and increases calcium signaling, including calpain activation. The involvement of the mitochondrial apoptotic pathway is supported by the decrease in mitochondrial membrane potential, down-regulation of anti-apoptotic Bcl-2 proteins, and activation of caspase 9. Subsequent activation of caspase 3 occurs after mitochondrial damage, leading to PARP-1 cleavage and resulting in apoptotic cell death (Figure 3).

### 4.1. ER Stress in Cancer

Tumor cells are frequently subjected to microenvironmental stressors such as hypoxia, nutrient deprivation, acidosis, and redox imbalance, all of which disrupt endoplasmic reticulum function and lead to chronic activation of the UPR [88,89]. While acute ER stress may result in cell death, cancer cells often hijack the adaptive arms of the UPR to support survival, metabolic flexibility, and progression [90].

Persistent ER stress has been shown to promote oncogenesis by facilitating epithelial-to-mesenchymal transition (EMT), angiogenesis, and invasion [91]. Glucose-regulated proteins (GRPs), particularly GRP78, GRP94, GRP170, and GRP75, are overexpressed in many tumor cell lines and are associated with aggressive growth and invasive properties. These proteins play critical roles in cancer by regulating the balance between cell survival and cell death, facilitating the maturation of oncogenic factors such as insulin-like growth factor 1 (IGF-1), Toll-like receptors (TLRs), integrins, and vascular endothelial growth factor A (VEGFA), and interacting with the tumor suppressor p53 to block its pro-apoptotic activity [92,93].

ER stress and therapy resistance induce the expression of GRP78 on the cell surface, where it activates the oncogenic phosphoinositide 3-kinase–protein kinase B (PI3K–AKT) signaling pathway. Murine models with conditional deletion of Grp78 and phosphatase and tensin homolog (Pten)—a tumor suppressor that antagonizes PI3K—demonstrate that GRP78 is required for the full activation of PI3K–AKT signaling, highlighting its role as a co-factor in prostate tumorigenesis and leukemogenesis. Moreover, GRP78 has been identified as a downstream target of the IGF-1 receptor–PI3K (IGF-1R–PI3K) signaling pathway, suggesting the existence of proliferative feedback loops between mitogenic signaling and ER stress [91,94].

PERK-eIF2α-ATF4 signaling, for instance, enhances tumor cell resilience by modulating amino acid metabolism, redox balance, and autophagy [95]. In parallel, IRE1α–XBP1s contributes to lipid biosynthesis and secretion, supporting tumor growth and interaction with the microenvironment [96].

Numerous lines of evidence indicate that the integrated stress response (ISR), a branch of the unfolded protein response, is activated under hypoxic and anoxic conditions both in vitro and in vivo, contributing to cellular adaptation to oxidative stress and tumor cell survival. The inability of cancer cells to effectively activate the ISR under hypoxia results in increased apoptosis, reduced cellular viability, and impaired tumor growth. In particular, the IRE1–XBP1 signaling axis has been shown to be essential for survival under hypoxic conditions [97].

During hypoxia, despite global translational repression, the selective expression of key genes such as ATF4, CHOP, BiP, and ORP150 is maintained, likely due to structural features of their mRNAs (e.g., complex 5′ untranslated regions). ATF4 not only regulates downstream UPR genes, but also contributes to the antioxidant defense, suggesting a functional interplay between oxidative and endoplasmic reticulum stress. In contrast, CHOP, though induced by ATF4, exhibits pro-apoptotic activity, and its prolonged activation may promote cell death in conditions of severe or sustained hypoxia [95].

Altogether, these findings support the notion that the UPR plays a critical role in hypoxia tolerance and tumor progression. Given that severe hypoxia is a hallmark of the tumor microenvironment, pharmacological targeting of UPR components may offer a selective therapeutic strategy against hypoxia-adapted cancer cells.

Importantly, ER stress also plays a central role in immune evasion. Lipid peroxidation, through metabolites such as 4-hydroxynonenal (4-HNE), induces ER stress and constitutively activates the IRE1α–XBP1 signaling axis, promoting aberrant lipid accumulation in tumor-infiltrating dendritic cells (tDCs) and impairing antigen presentation. This process compromises T cell–mediated anti-tumor immunity. Targeted deletion of XBP1 in tDCs, or its therapeutic silencing using siRNA-loaded nanocarriers, restores type 1 immune responses and improves survival in murine models of ovarian cancer (OvCa) [98].

Tumor cells undergoing ER stress have also been shown to transmit signals to recipient macrophages, inducing a pro-inflammatory and tumor-promoting state [99,100]. This phenomenon, referred to as transmissible ER stress, involves heat-resistant soluble factors, enhanced by lipopolysaccharide (LPS) and sensed via Toll-like receptor 4 (TLR4), suggesting a mechanistic link between inflammation and ER stress within the tumor microenvironment. The conditioned macrophages exhibit an inflammatory transcriptional signature, characterized by increased expression of Il-6, Il-23p19, and Tnf-α, but do not regulate classical M1/M2 markers, indicating an atypical inflammatory myeloid phenotype. The IRE1α/XBP1 pathway may mediate this effect, similarly to what has been observed in adipose tissue-associated macrophages. Furthermore, the upregulation of Il-23p19, known to suppress T cell and natural killer (NK) cell functions, may contribute to the inhibition of anti-tumor immunity and promote the differentiation of regulatory T cells (Tregs) and Th17 cells, thereby expanding inflammation [100].

Another critical aspect of ER stress in cancer is its contribution to chemoresistance. High UPR activity has been correlated with poor prognosis and treatment failure in several malignancies, including triple-negative breast cancer, pancreatic ductal adenocarcinoma, and hepatocellular carcinoma [101,102].

These findings underscore the therapeutic potential of targeting ER stress in cancer. Strategies include the use of small-molecule inhibitors of IRE1α RNase activity or PERK kinase function, as well as agents that selectively overload ER stress to push tumor cells beyond their adaptive capacity [103]. Additionally, immunotherapeutic combinations with UPR modulators are being investigated to improve antitumor immunity and reverse immune suppression in the tumor microenvironment [104].

ER stress in cancer is not merely a passive response to environmental stress, but a dynamic driver of tumor progression, immune modulation, and therapeutic resistance. Its selective targeting may offer novel opportunities for cancer therapy, particularly in tumors with high proteostatic or metabolic stress burdens.

### 4.2. Lysosomal Dysfunction and ER Stress in Cancer

UPR signaling balances tumor cell survival and death, influencing therapeutic resistance and apoptosis. During tumorigenesis, UPR maintains ER protein folding and proteostasis, but if ER stress is unresolved, apoptosis can be triggered, often exacerbated by lysosomal dysfunction [105,106].

It has already been discussed how the lysosomal autophagic pathway in tumor cells is mediated by UPR transducers; thus, there could be a close correlation between lysosomes and the endoplasmic reticulum. It follows that, if lysosomes do not function properly due to genetic lysosomal dysfunctions, lysosomal diseases, or other disorders that compromise their integrity or enzymatic activity, this can interfere with the cells’ ability to complete the autophagic process. Consequently, autophagy may not occur effectively or may be reduced, with potential negative consequences for tumor survival [107].

In a study by Yu et al., it was found that reduced autophagic flux by NH_4_Cl increased the sensitivity of HeLa cells to menadione [108]. Menadione could lead to the accumulation of ubiquitinated proteins and increase the expression of GRP78 protein, thereby activating ER stress and autophagy in human cervical cancer cells. By blocking the lysosomal system, the cells’ ability to survive OS is compromised, allowing the UPR to activate apoptosis. Thus, lysosomal dysfunction could be a therapeutic target to enhance the efficacy of menadione.

Numerous studies have highlighted that the increase in lysosomes necessary for autophagy and the regulation of membrane-bound LC3-II expression occur during ER stress in various types of tumors. In a study on the silencing of IRE1α–PERK–ATF6 axis during ER stress, autophagy was specifically suppressed in the IRE1α–TRAF2–JNK signaling pathway [109]. Interestingly, ER stress-induced autophagy can inhibit tumor progression, but also protect tumor cells. In studies on breast cancer cells, tunicamycin increases the UPR, thus activating the protective mechanism mediated by ER stress through the IRE1/JNK/beclin-1 pathway. The inhibition of ER stress or activation of JNK leads to the inhibition of autophagy-mediated apoptosis [110].

In studies on BC3 tumor cells, ER stress-induced autophagic flux is regulated via the PERK–eIF2α–CHOP pathway [111]. It has been shown that polyQ72 aggregates can increase the expression of autophagic proteins ATG12 and CHOP to promote autophagy, while the inhibition of eIF2α phosphorylation can reduce their expression, indicating the involvement of the PERK pathway in ER stress-induced autophagy.

In neuroblastoma, ER stress causes mitochondrial dysfunction by activating p-eIF2α, while the B-Raf inhibitor PLX4720 upregulates the PERK pathway in melanoma to stimulate ER stress-induced autophagic flux [112]. Additionally, tumor cells lacking the ATG5 gene, a protein involved in the early stage of autophagosome formation, show an increased response to ER stress, suggesting that the inhibition of autophagy can intensify ER stress in tumor cells and induce apoptosis [113].

In another study, it was demonstrated through genetic inhibition that the suppression of autophagy has an anticancer effect on colorectal cancer in Atg5 mutant mice treated with azoxymethane/dextran sodium sulfate (AOM/DSS) [114]. The suppression of autophagy caused antitumor effects via apoptosis induced by prolonged activation of the UPR.

Much evidence has demonstrated that inducing autophagy in cancer can be a useful treatment strategy. In a study on melanoma, the small molecule HA15 induced apoptosis and autophagy by targeting GRP78 [115]. During this process, vesicle aggregation, the conversion of LC3-I to LC3-II, and the formation of autophagosomes were observed. The therapeutic efficacy of HA15 on melanoma cells decreased when autophagy and apoptosis were inhibited, suggesting that autophagy can inhibit tumor growth. The study on HA15 demonstrated that, when apoptosis was inhibited (using QVD, a caspase inhibitor) and autophagy was blocked (with siLC3, which silences LC3B), both apoptosis and autophagy markers, such as PARP cleavage and LC3B expression, were reduced. However, CHOP expression and ER stress markers (IRE1α) remained unaffected, showing that CHOP’s activity remains essential for stress signaling, independent of apoptosis or autophagy inhibition. When both autophagy and apoptosis were blocked simultaneously, cell viability was fully restored, indicating that these pathways work together under CHOP’s regulation to induce cell death in response to HA15. Thus, CHOP was crucial for both autophagy and apoptosis in HA15-induced ER stress, because it mediates stress signaling that trigger both pathways, leading to melanoma cell death. This dual role is due to CHOP’s ability to regulate genes involved in both cell death and survival mechanisms.

One of the hallmarks of cancer cells is their resistance to apoptosis. Consequently, numerous studies have focused on overcoming this resistance by targeting pathways that lead to apoptosis. LMP often precedes apoptosis in response to cytotoxic compounds in cancer cells.

It has been reported that there is an overexpression of the Sp1 protein in colon, gastric, pancreatic, and breast cancers, unlike in normal cells where there is minimal or no expression. Sp1 is a transcription factor that regulates many biological functions, including cell growth, differentiation, survival, tumor progression, and metastasis. Because of its binding sites present in a large number of genes, it is likely that the downregulation of Sp1 would have a profound effect on the survival of cancer cells. Moreover, Sp transcription factors (Sp1, Sp3, and Sp4) are constitutively bound to the ER stress response element and are essential for gene regulation in response to ER stress [116,117].

Based on in vitro studies on pancreatic cancer cells (MIA PaCa-2) and in vivo studies on athymic nude mice treated with the same cells, it was found that, after Sp1 silencing and the induction of stress via TM, Sp1 does not bind to the Grp78 promoter [118]. Without active transcription of Grp78, the stress threshold that cells can manage is exceeded, leading to cell death. Therefore, the downregulation of Sp1 in pancreatic cancer results in chronic ER stress, which in turn leads to a sustained increase in cytosolic calcium, LMP, and cell death.

The connection point between chronic ER stress activation and death induction due to LMP is likely due to the alteration of cytoplasmic calcium regulation. We know that prolonged ER stress leads to calcium accumulation in cells; thus, Sp1 downregulation leads to chronic ER stress and prolonged calcium accumulation, which activates LMP. Therefore, the Sp1 protein could be considered one of the protective factors in cancer.

Bortezomib, a proteasome inhibitor, disrupts ER proteostasis by blocking protein degradation pathways, leading to ER stress and UPR activation. This increased stress can elevate calcium influx, impacting lysosomal membrane stability and promoting LMP, which enhances apoptosis [119]. Kao et al., in their study, suggest that bortezomib interrupts the autophagic process in cancer cells without preventing the merging of autophagosomes with lysosomes. In particular, in ovarian, endometrial, and hepatocellular carcinoma cells, bortezomib hindered lysosomal protein degradation by reducing the activity of cathepsins [120]. Another drug, chloroquine, inhibits lysosomal acidification, which blocks autophagic flux, indirectly escalating ER stress and inducing apoptosis in cancer cells under oxidative conditions [121].

On the other hand, the study by Yu et al. highlights how macrolide antibiotics, such as azithromycin (AZD), impact cancer cells by inhibiting lysosomal acidification, which disrupts autophagic flux and triggers ROS accumulation. This ROS build-up leads to ER stress and activates the phosphorylation of eIF2α and downstream pathways like ATF4 and CHOP signaling. This cascade results in the translocation of transcription factors TFEB and TFE3, crucial for inducing autophagy and regulating OS. Interestingly, these pathways, while blocking autophagy, enable moderate ROS levels, which paradoxically support tumor survival and proliferation through mechanisms like tumor cell dormancy and immune evasion [122].

Lysosomal membrane contact sites (MCSs) play a critical role in regulating cellular homeostasis, but their dysfunction contributes to cancer progression [123]. The ER–lysosome MCS influences cholesterol and lipid trafficking, impacting mTORC1 signaling, which is often hyperactivated in cancer to promote growth and metabolic adaptation. Disruptions in cholesterol transfer, as seen in Niemann Pick type-C 1 (NPC1) protein mutations, lead to constitutive mTORC1 activation, fostering uncontrolled proliferation [124]. Regarding cholesterol transport, the START domain 3 (STARD3) protein facilitates the formation of endosome–ER contact sites, leading to an increased buildup of cholesterol in endosomes while reducing its availability at the plasma membrane. Perretti et al. demonstrated that the overexpression of STARD3 disrupts endosome maturation by creating static ER–endosome contact sites, preventing their transition to lysosomes and blocking receptor degradation. This leads to increased recycling of HER2 and growth factor receptors, amplifying oncogenic signaling and promoting HER2-positive cancer progression [125]. Additionally, the ER–lysosome MCS regulate Ca^2+^ exchange, which, when impaired, prevents Ca^2+^-mediated apoptosis, allowing cancer cells to evade cell death [126,127]. The lysosome–mitochondria MCS plays a key role in mitochondrial dynamics and calcium transfer, but its uncoupling in cancer cells reduces Ca^2+^ flux, promoting survival and metastatic potential. Overall, cancer cells hijack the lysosomal MCS to manipulate signaling pathways, evade immune responses, and enhance their invasive and metastatic capabilities, making them crucial targets for novel therapeutic strategies [128].

Therefore, the UPR signaling pathway is crucial for balancing tumor cell death and growth, affecting chemotherapeutic sensitivity and apoptosis induction. Through UPR activation, ER protein folding capacity is preserved, maintaining proteostasis during tumorigenesis and typically counteracting apoptosis. However, when ER stress is severe or prolonged, as in cases of lysosomal dysfunction, apoptosis can proceed. This connection between lysosomes and the ER becomes especially important, as UPR transducers (PERK, IRE1, and ATF6) coordinate with the lysosomal autophagic pathway in cancer cells to maintain homeostasis. If lysosomal function is compromised, for example, by enzymatic activity disruptions, autophagy efficacy decreases, affecting tumor cell survival by failing to clear damaged proteins.

The tight functional coupling between the ER and lysosomes is further reinforced by their shared engagement in responding to diverse forms of cellular stress, forming a broader stress-response triad. While OS plays a central role in this interplay, it acts in concert with other tumor-associated stressors, including hypoxia, nutrient deprivation, metabolic reprogramming, and genotoxic insults.

Hypoxia, a hallmark of solid tumors, stabilizes hypoxia-inducible factor 1-alpha (HIF-1α) and activates adaptive responses such as autophagy, UPR, and metabolic shifts. The resulting ROS accumulation amplifies ER stress and promotes TFEB nuclear translocation, enhancing lysosomal biogenesis and autophagic flux. This hypoxia-driven axis contributes to tumor adaptation and therapy resistance [129,130]. Nutrient deprivation, especially that of glucose and amino acids, suppresses mTORC1 and activates AMPK, inducing autophagy and lysosomal biogenesis via TFEB and TFE3. It also alters ER–lysosome contact sites, affecting calcium and lipid signaling. Prolonged starvation impairs autolysosome formation and exacerbates proteostasis imbalance [131,132]. Metabolic stress, linked to mitochondrial dysfunction or oncogenic mutations (e.g., v-myc avian myelocytomatosis viral oncogene homolog (MYC) and Kirsten rat sarcoma viral oncogene homolog (KRAS)), increases ROS and disrupts calcium homeostasis, triggering ER stress via the PERK–eIF2α–ATF4 and IRE1–JNK–CHOP pathways. Simultaneously, impaired mitophagy hinders lysosomal clearance, aggravating cellular stress and fostering malignancy [68,133]. These stressors converge on shared signaling nodes, such as calcineurin–TFEB, AMPK–ULK1, and PERK–ATF4, which orchestrate lysosomal and ER responses. Their dynamic interplay dictates whether cancer cells adapt or undergo cell death.

The convergence of lysosomal dysfunction, ER stress, and OS constitutes a critical signaling hub in cancer biology. Although these pathways are mechanistically distinct, they often co-activate in response to oncogenic stress, modulating cell fate decisions, such as adaptation, survival, or apoptosis. The ER governs protein folding, lipid synthesis, and calcium homeostasis, whereas lysosomes coordinate degradation via autophagy and endocytosis. Under pathologic conditions, both organelles become overwhelmed, leading to the accumulation of misfolded proteins, reactive oxygen species, and proteotoxic aggregates, which can trigger the UPR and LMP. The extent and directionality of these responses are highly context-dependent, influenced by tumor origin, genetic background, microenvironmental conditions, and metabolic demands.

Across tumor types, distinct patterns emerge in how these stress pathways are activated and exploited (Table 1).

In melanoma, high basal ER stress and ROS levels are typical. Cathepsin L inhibitors induce LMP and IRE1-mediated apoptosis, with NLRP3 inflammasome activation linking lysosomal and redox stress [14,46,134,135].

Breast cancer subtypes, including triple-negative tumors, preferentially exploit the adaptive arms of the UPR (e.g., GRP78 and XBP1s) to support survival. Autophagy defects and sustained ROS contribute to therapy resistance [80,136,137].

In glioblastoma, ER stress (via ATF6 and PERK) is activated under hypoxia and DNA damage, while lysosomal impairment and mitochondrial ROS sustain tumor cell viability [138,139].

Pancreatic cancer is marked by chronic ER stress and hypoxia-induced ROS, promoting cathepsin-dependent LMP and sensitizing cells to ER-targeted apoptosis [95,118].

In hepatocellular carcinoma, the full spectrum of UPR branches (PERK, IRE1, and ATF6) is active. Persistent ER stress, impaired autophagy, and ROS accumulation converge with ferroptotic signaling [140,141,142].

In acute myeloid leukemia (AML), chemotherapeutic stress activates UPR transcription factors (e.g., XBP1 and ATF4), while lysosomal adaptation supports survival and ROS enhances genomic instability [99,143].

Recent data also indicate that nanomaterials can amplify this triad by inducing lysosomal destabilization and subsequent ER stress in a tumor-specific manner, reinforcing its therapeutic relevance [135,136,144,145].

These tumor-specific signatures underscore the dynamic plasticity of the ER–lysosome–OS axis. In aggressive cancers such as melanoma and HCC, this network is deeply embedded in tumor progression, whereas in others, like breast cancer and leukemia, it is more selectively engaged. Understanding these distinctions can inform the development of personalized strategies targeting these stress nodes.

## 5. Future Perspectives: Targeting Lysosomes and ER in Cancer Therapy

Targeting the functional alterations of lysosomes and the ER in cancer represents a rapidly evolving and promising therapeutic strategy. Both organelles are deeply involved in tumor progression, stress adaptation, and resistance to therapy, and their dysregulation creates vulnerabilities that can be selectively exploited. A summary of key molecular pathways involved in ER and lysosomal stress responses, along with their modulators and therapeutic status, is provided in Table 2.

Lysosomes, beyond their role in cellular waste degradation, are actively repurposed by cancer cells to support anabolic processes, sustain autophagy under nutrient stress, and resist chemotherapeutic damage. Conventional autophagy inhibitors, such as chloroquine and hydroxychloroquine, have been explored to interfere with lysosomal acidification and autophagic flux. Although these agents have demonstrated some efficacy, their broad effects and limited potency have raised concerns about toxicity and off-target consequences [68,145,146,147]. More recently, novel lysosomal inhibitors such as dimeric quinacrines or palmitoyl-protein thioesterase 1 (PPT1) inhibitors have shown the ability to disrupt multiple lysosome-dependent pathways, including mTORC1 signaling and lysosomal enzyme activity, while inducing LMP. This leads to the release of proteases such as cathepsins into the cytosol, initiating apoptotic and inflammatory responses. Importantly, these agents can trigger immunogenic cell death (ICD), a form of regulated cell death characterized by the release of damage-associated molecular patterns (DAMPs) like ATP, HMGB1, and calreticulin exposure, which stimulate dendritic cell maturation and cytotoxic T cell responses against the tumor [148].

Nanotechnology further enhances this approach by allowing the design of pH-sensitive nanoparticles that selectively deliver cytotoxic drugs to the acidic lysosomal compartments of tumor cells, improving drug specificity and reducing systemic toxicity [149,150].

On the other hand, the ER plays a central role in protein folding and calcium homeostasis. Cancer cells, due to their high metabolic and biosynthetic demands, experience chronic ER stress and rely heavily on the UPR to maintain viability. Therapeutic agents that interfere with this balance aim either to inhibit adaptive UPR pathways, such as PERK, IRE1α, or ATF6, or to exacerbate ER stress to cytotoxic levels. Thapsigargin analogs and proteasome inhibitors like bortezomib act by blocking protein clearance mechanisms and inducing the accumulation of misfolded proteins, ultimately driving cells toward apoptosis [119,151,152]. These effects are amplified when ER-targeting agents are combined with lysosomal inhibitors, producing a dual blockade of degradation pathways (autophagy and ER-associated degradation), resulting in metabolic collapse and death of cancer cells [153,154,155,156].

**Table 2:** Summary of key stress pathways, molecular modulators, and implications in cancer.

Pathway/Target	Function in Stress Response	Modulating Compounds	Effect in Cancer	Clinical Status
PERK–eIF2α–ATF4	UPR activation, autophagy, apoptosis	GSK2606414, ISRIB	Pro-apoptotic/ cytoprotective	Preclinical [106,151,153,154]
IRE1–XBP1/ IRE1–JNK	UPR signaling, autophagy, apoptosis	4μ8C, MKC8866	Mixed: context-dependent	Preclinical/Clinical trial [80,111,155]
TFEB (via calcineurin/ROS)	Lysosomal biogenesis, autophagy induction	Genistein, trehalose	Enhances autophagy/proteostasis	Preclinical/Repurposing [26,136,147]
mTORC1	Autophagy suppression, growth promotion	Rapamycin, Everolimus (rapalogs)	Tumor growth inhibition	Approved [58,156]
AMPK	Induces autophagy, inhibits mTORC1	Metformin, AICAR	Metabolic reprogramming, ROS balance	Approved/Clinical trial [62,63]
LMP	Induces apoptosis, inflammation	Siramesine, Apoptozole	Triggers lysosome-mediated death	Preclinical [70,71]
Cathepsins	ECM degradation, apoptosis, immune evasion	E64d, CA-074, CTSB inhibitors	Tumor invasion/resistance modulation	Preclinical [13,46]
ROS modulation	Triggers ER and lysosomal stress	Bortezomib, HA15, Chloroquine	Sensitizes cells to stress	Approved/Clinical trial [115,119,120,131]

Despite these promising advances, several unanswered questions remain. A major goal is to identify predictive biomarkers that could guide the use of ER- and lysosome-targeted therapies in personalized oncology. Furthermore, the complex crosstalk between these organelles and their integration into tumor-specific signaling networks must be further elucidated. Future studies should focus on developing selective and less toxic compounds that precisely modulate organelle function. Finally, integrating these strategies with immunotherapy, leveraging the immunogenic potential of organelle-targeted stress and death pathways, could represent a powerful combinatorial approach to overcome resistance and enhance long-term tumor control.

## 6. Conclusions

The intricate crosstalk between OS, ER stress, and lysosomal dysfunction represents a fundamental mechanism underlying cancer development, progression, and resistance to therapy. Under physiological conditions, ROS serve as critical signaling molecules maintaining cellular homeostasis. However, excessive ROS production disrupts protein folding, induces ER stress, destabilizes lysosomal membranes, and leads to LMP, causing the release of calcium, iron, and hydrolytic enzymes that amplify cellular damage.

In cancer cells, which are characterized by chronic oxidative stress due to increased metabolic activity, these mechanisms are profoundly altered. Lysosomal dysfunction is exploited to support tumor survival, with changes in lysosomal number, localization, and activity facilitating macromolecule degradation, recycling, and adaptation to the hostile tumor microenvironment. Moreover, impaired mitophagy further increases ROS levels, fueling tumor progression. Lysosomal enzyme release following lysosomal hyperacidification does not lead to cell death as in normal cells, but instead promotes invasion, metastasis, and chemoresistance.

ER stress similarly exhibits a dual role in cancer: while acute ER stress can activate adaptive mechanisms such as autophagy and the UPR to restore homeostasis, chronic or excessive ER stress can lead either to apoptosis or enhanced tumor survival, depending on the modulation of pathways like PERK, IRE1, and ATF6. In early tumorigenesis, ER stress-induced autophagy allows cancer cells to manage oxidative stress and damaged components, thus favoring tumor growth. In contrast, failure of lysosomal degradation can shift ER stress responses toward apoptosis in advanced stages.

Importantly, cancer cells co-opt lysosomal and ER stress signaling to resist therapy. The overexpression of lysosomal enzymes such as cathepsin B, regulated by oncogenic pathways like HER2 signaling, exemplifies the integration of ER stress responses and lysosomal function to promote metastatic potential and therapeutic resistance. Additionally, alterations in autophagy, CMA, and microautophagy impact the degradation of oxidized proteins and lipids, exacerbating oxidative stress when these processes are suppressed.

In light of these findings, targeting the stress adaptation mechanisms of cancer cells represents a promising therapeutic avenue. Pharmacological modulation of lysosomal activity, autophagy, and ER stress pathways could selectively induce cancer cell death by overwhelming their stress tolerance capacity. Nevertheless, due to the context-dependent roles of these pathways, therapeutic strategies must be carefully designed to prevent unintended promotion of tumor survival. Future research should focus on elucidating the specific regulatory networks involved and identifying precise molecular targets to maximize anticancer efficacy while minimizing off-target effects.

## Figures and Tables

**Table 1:** Comparative summary of endoplasmic reticulum (ER) stress, lysosomal dysfunction, and oxidative stress (OS) across tumor types. The symbol ↑ indicates increased activity, expression, or levels of the indicated factor or process.

Tumor Type	ER Stress Pathways	Lysosomal Alterations	OS Role	Key Findings/Effects
Melanoma	IRE1, PERK, CHOP	LMP, cathepsin release and LC3-II accumulation	↑ ROS, NLRP3 activation	Cathepsin L inhibition induces ER-stress-mediated apoptosis [14,46,134,135]
Breast cancer	GRP78, XBP1s	Defective autophagy	Moderate ROS supports survival	UPR–autophagy axis confers chemoresistance [80,136,137]
Glioblastoma	ATF6, PERK	Impaired lysosomal clearance	↑ Mitochondrial ROS	ER–mitochondria–lysosome crosstalk sustains viability [138,139]
Pancreatic cancer	CHOP, ATF4	LMP and cathepsin release	↑ ROS sensitizes cells	ER–lysosome synergy promotes apoptosis [95,118]
Hepatocellular carcinoma	PERK, IRE1, ATF6	Compromised autophagy	↑ ROS, ferroptosis involvement	Chronic ER stress supports tumor growth [140,141,142]
Myeloid leukemia (AML)	XBP1, ATF4	Adaptative autophagy	↑ ROS promotes mutagenesis	UPR targeting enhances chemosensitivity [99,143]

## Data Availability

No new data were created or analyzed in this study.
